# Antiphotoaging Effect of AGEs Blocker™ in UVB-Irradiated Cells and Skh:HR-1 Hairless Mice

**DOI:** 10.3390/cimb45050266

**Published:** 2023-05-09

**Authors:** JaeIn Jung, Yean-Jung Choi, JinHee Yoo, Su-Young Choi, EunJi Kim

**Affiliations:** 1 Industry Coupled Cooperation Center for Bio Healthcare Materials, Hallym University, Chuncheon 24252, Republic of Korea; dlswowjd@naver.com; 2 Department of Food and Nutrition, Sahmyook University, Seoul 01795, Republic of Korea; yjchoi@syu.ac.kr; 3 Functional Ingredient Development Team, COSMAX NS, INC., Seongnam-si 13486, Republic of Korea; yoojinhee@cosmax.com; 4 Functional Ingredient Development Team, COSMAX NBT, INC., Seongnam-si 13486, Republic of Korea; sychoifwp@cosmax.com

**Keywords:** UVB, antiphotoaging, Korean mint, fig, goji berry, AGEs Blocker^TM^

## Abstract

Chronic exposure to ultraviolet (UV) radiation is a major cause of photoaging. It involves extrinsic aging, wrinkle formation, and skin dehydration, and leads to excessive production of active oxygen that adversely affects the skin. Here, we investigated the antiphotoaging effect of AGEs Blocker^TM^ (AB), which comprises Korean mint aerial part and fig and goji berry fruits. Compared to its individual components, AB was more potent at increasing the expression of collagen and hyaluronic acid and decreasing MMP-1 expression in UVB-irradiated Hs68 fibroblasts and HaCaT keratinocytes. In Skh:HR-1 hairless mice exposed to 60 mJ/cm^2^ UVB for 12 weeks, oral administration of 20 or 200 mg/kg/day AB restored skin moisture by improving UVB-induced erythema, skin moisture, and transepidermal water loss, and alleviated photoaging by improving UVB-induced elasticity and wrinkles. Moreover, AB upregulated the mRNA levels of hyaluronic acid synthase and collagen-related *Col1a1*, *Col3a1*, and *Col4a1* genes, increasing hyaluronic acid and collagen expression, respectively. AB inhibited UVB-induced MAPK and AP-1 (c-fos) activation, resulting in significantly downregulated expression of MMP-1 and -9, which are responsible for collagen degradation. AB also stimulated the expression and activity of antioxidative enzymes and reduced lipid peroxidation. Thus, AB is a potential preventive and therapeutic agent for photoaging.

## 1. Introduction

Skin is the first line of defense against external environmental factors, safeguarding the human body against harmful environmental stressors, such as pathogens, chemicals, air pollutants, and solar ultraviolet (UV) radiation [1]. Skin aging is caused by internal and external factors, which induce chronological (natural) skin aging that occurs with the passage of time, and extrinsic aging (or photoaging). UV irradiation is a major factor in skin photoaging [2], which can damage the skin by altering its cellular composition and organization and causing loss of the extracellular matrix (ECM), resulting in the occurrence of roughness, relaxation, wrinkles, and pigmentation [2,3,4,5].

Excessive exposure to UV radiation, especially UVB (λ = 280–320 nm), generates reactive oxygen species (ROS), which is recognized as the main cellular factor in physiological photoaging [6]. The production of high levels of ROS induced by UVB leads to activation of the mitogen-activated protein kinase (MAPK) signaling pathway, and subsequently that of transcription factors, such as activator protein (AP)-1 and nuclear factor-κB (NF-κB). These transcription factors regulate the transcription of genes responsible for the degradation of the ECM, such as matrix metalloproteinases (MMPs). In addition, they are linked to several alterations in the molecular signaling pathway in the dermis and epidermis, which lead to disrupted antioxidative systems, pigmentation, and skin dryness [6,7,8,9].

Korean mint (*Agastache rugosa* O. Kuntze) is a perennial plant in the Labiate family, mostly found in Korea and Northeast Asia. It has been widely used as a spice and traditional medicine [10]. Korean mint extract has various physiological benefits and exhibits antiatherosclerotic [11], antioxidative [12,13], and anti-inflammatory effects [14]. It has been reported to exert antiphotoaging effects in UVB-irradiated human keratinocytes [15,16] and UVB-irradiated hairless mice [17]. Goji berry (*Lycium barbarum*), a dried fruit of the goji berry tree in Solanaceae, which is distributed in Korea and China, is widely used as a traditional medicine [18]. It has been reported to have immune-enhancing [19] and hepatoprotective [20] properties, and was shown to inhibit nonenzymatic glycation in an aging mouse model [21]. The fig (*Ficus carica* L.) is a tree belonging to the Moraceae family, which is mainly cultivated in the Middle East and Mediterranean regions. Its delicious and nutritious fruit has been used since ancient times [22]. Various fig extracts are known for their health-promoting and disease-protective properties including antioxidative [23], cardioprotective [24], hepatoprotective [25], and hypoglycemic [26] effects. In a previous study, the hot water extract of a mixture consisting of Korean mint aerial part (KE), fig fruits (FE), and goji berry fruits (GE) was shown to exhibit strong DPPH radical-scavenging and tyrosinase-inhibiting activities. Furthermore, this extract inhibited the expression of MMP-1 in HaCaT cells and increased the production of type I collagen in Hs68 cells [27], which are all indicative of its potential in alleviating skin photoaging.

Based on these research reports, the mixed ingredient was expected to show the synergistic effect compared to each ingredient, such as KE, FE, and GE, against skin aging. Therefore, in the present study, to confirm this synergistic effect of the mixed ingredient, we investigated the antiphotoaging effect of the standardized aqueous extract of a mixture consisting of KE, FE, and GE (AGEs Blocker^TM^, AB) and compared it with that of the individual components. We first determined the effects of AB on collagen, hyaluronic acid, and MMP-1 expression in skin fibroblasts and keratinocytes. Subsequently, we also examined the effects of oral administration of AB on UVB irradiation-induced skin photoaging and investigated the molecular mechanisms underlying the effects of AB in UVB-irradiated Skh:HR-1 hairless mice. We show that AB inhibits photoaging more potently than any of its individual components.

## 2. Materials and Methods

### 2.1. Preparation of Extracts and High-Performance Liquid Chromatography (HPLC) Analysis

The aqueous extracts of KE, FE, and GE and the standardized aqueous extract, AB, used in this study were procured from COSMAX NS, INC. (Seongnam, Korea). In brief, the dried raw materials were extracted at 95 °C for 4 h, and the extract was filtered. Each filtrate was concentrated by vacuum evaporation, mixed with maltodextrin, and dried by spray-drying to obtain each extract. The HPLC system used to analyze the content of tilianin, a bioactive compound, was an Agilnet (Agilent Technologies, Inc., Santa Clara, CA, USA) 1290 II series. The HPLC column was Sunfire C18 (250 × 4.6 mm, 5 μm, Waters Corp., Milford, MA, USA). The mobile phase was (A) water at 0.1% formic acid and (B) acetonitrile. The gradient started with 10% B and was varied to 10% B at 5 min, 40% B at 25 min, 70% B at 40 min, 90% B at 45 min, 10% B at 45.1 min, and 10% B isocratic from 45.1 to 50 min. Analysis wavelength was 330 nm, flow rate was 1.0 mL/min, column oven temperature was maintained at 35 °C, and sample injection rate was 10 μL.

### 2.2. Cell Culture

Hs68 human skin fibroblasts were obtained from the American Type Culture Collection (ATCC, Manassas, VA, USA). HaCaT human skin keratinocytes were purchased from CLS Cell Lines Service GmbH (Eppelheim, BW, Deutschland). Hs68 and HaCaT cells were cultured in Dulbecco’s modified Eagle’s medium (DMEM) supplemented with 10% fetal bovine serum (FBS), 100,000 U/L penicillin, and 100 mg/L streptomycin at 37 °C in a humidified atmosphere with 5% CO_2_.

### 2.3. Quantitation of Collagen and Matrix Metalloproteinase (MMP)-1 Production in Hs68 Cells

Hs68 cells were plated at a density of 1 × 10^5^ cells/well in 24-well plates and cultured for 24 h. Thereafter, the cells were rinsed twice with phosphate-buffered saline (PBS) and exposed to UVB radiation (30 mJ/cm^2^, 312 nm) using a UV-irradiation system (BIO-SUN, Vilber Lourmat, Marne la Vallee, France). After UVB irradiation, the cells were incubated in fresh serum-free DMEM in the presence or absence of 200 μg/mL KE, FE, GE, or AB and further incubated for 24 h. The cell culture media conditioned for 24 h were collected. The content of collagen and MMP-1 in the 24 h conditioned media was measured using a Procollagen Type I C-Peptide EIA kit (Takara Bio, Kusatsu, Japan) and human MMP-1 ELISA kit (Sigma-Aldrich, St. Louis, MO, USA), respectively, according to the manufacturer’s instructions.

### 2.4. Measurement of Hyaluronic Acid Production in HaCaT Cells

HaCaT cells were plated at a density of 1 × 10^5^ cells/well in 24-well plates and incubated for 24 h. Thereafter, the cells were rinsed twice with PBS and exposed to UVB radiation (5 mJ/cm^2^, 312 nm) using the UV irradiation system (Vilber Lourmat, Marne-la-Vallée, France). After UVB irradiation, the cells were incubated in fresh serum-free DMEM in the presence or absence of 200 μg/mL KE, FE, GE, or AB and further incubated for 24 h. The cell culture media conditioned for 24 h were collected. The content of hyaluronic acid in the 24 h conditioned media was measured using a Hyaluronan Quantikine ELISA kit (R&D Systems, Minneapolis, MN, USA) according to the manufacturer’s instructions.

### 2.5. Ethical Statement and Animal

All animal experiments were conducted in accordance with protocols approved by the Institutional Animal Care and Use Committee of Hallym University (Hallym 2022-9). Male, five-week-old Skh:HR-1 hairless mice were purchased from DooYeol Biotech Co., Ltd. (Seoul, Republic of Korea). They were kept under controlled standard conditions (23 ± 3 °C, 50 ± 10% relative humidity, and 12 h light/dark cycles) in the animal research facility of Hallym University. The mice were provided with a commercial non-purified rodent diet and tap water ad libitum.

### 2.6. Experiment Design and Treatment

After adaptation for one week, Skh:HR-1 hairless mice were randomly assigned to the following four groups (10 mice per group): (i) normal control group (without UVB irradiation, vehicle-administered group, NOR); (ii) UVB-irradiated control group (UVB irradiation, vehicle-administered group, UV); (iii) UVB-irradiated and 20 mg/kg body weight (BW)/day AB-administered group (UV+AB20); and (iv) UVB-irradiated and 200 mg/kg BW/day AB-administered group (UV+AB200). The mice in the UV+AB20 and UV+AB200 groups were orally administered AB (20 or 200 mg/kg BW/day) dissolved in 100 μL distilled water once daily for 12 weeks. The mice in the NOR and UV groups were orally administered an equal volume of distilled water as a vehicle. Before UVB irradiation, we determined the minimum erythema dose (MED) for dorsal skin, and 60 mJ/cm^2^ was determined to be 1 MED. All mice, except for those in the NOR group, were irradiated with UVB three times per week for 12 weeks, as follows: Week 1–2, 1 MED (60 mJ/cm^2^); Week 3–4, 2 MED (120 mJ/cm^2^); Week 5–6, 3 MED (180 mJ/cm^2^); Week 7–12, 4 MED (240 mJ/cm^2^). At the end of the experimental period, the mice were anesthetized with tribromoethanol, diluted in tertiary amyl alcohol. Dorsal skin samples were immediately taken for further analysis.

### 2.7. Histological Examination (H&E Staining)

Dorsal skin tissues were fixed in 4% paraformaldehyde immediately after removal. The fixed skin tissues were embedded in paraffin, sectioned to a thickness of 5 µm, deparaffinized with xylene, and rehydrated in a decreasing alcohol series up to distilled water. The tissue sections were stained with hematoxylin and eosin (H&E). The histological changes were examined and photographed under a light microscope (AxioImager, Carl Zeiss, Jena, Germany).

### 2.8. Assessment of the Indices Reflection the Skin Condition

One day before the end of the experiment, various indices were estimated to determine whether AB administration improved skin conditions. Skin erythema index was determined using Mexameter^®^ MX18 (Courage-Khazaka Electronic GmbH, Köln, Germany). Skin hydration index, transepidermal water loss (TEWL), and elasticity index were measured using Corneometer^®^ CM825, Tewameter^®^ TM300, and Cutometer^®^ MPA580 (Courage-Khazaka Electronic GmbH), respectively. To evaluate wrinkles, skin replicas were cast on the dorsal skin surface of mice using SILFLO (Flexico Developments Ltd., Tokyo, Japan) and measured using a Visionmeter SV600 (Courage-Khazaka Electronic GmbH). The topography of the skin surface was analyzed for total roughness (the distance between the highest peak and the lowest value, R1), average roughness (the average of the five maximum distances, R2), maximum roughness (the largest value of the five maximum distances, R3), smoothness depth (R4), and arithmetic average roughness (R5).

### 2.9. Quantitation of Hyaluronic Acid, Collagen, and MMPs in the Skin Tissue

Dorsal skin tissue samples were homogenized in PBS and centrifuged at 5000 rpm for 10 min. The supernatants were collected and used for enzyme-linked immunosorbent assay (ELISA). The protein content of the supernatant was assayed using a bicinchoninic acid (BCA) protein assay kit (Thermo Scientific, Rockford, IL, USA). The content of hyaluronic acid (R&D Systems, Minneapolis, MN, USA), collagen (Abcam, Cambridge, UK), MMP-1 (MyBioSource, San Diego, CA, USA), and MMP-9 (R&D Systems, Minneapolis, MN, USA) in skin homogenates was measured using the ELISA kits, according to the manufacturer’s instructions.

### 2.10. Measurement of Lipid Peroxidation and Antioxidative Enzyme Activity in the Skin Tissue

Dorsal skin tissue samples were homogenized in PBS and the resultant homogenates were collected. The protein content of the skin homogenates was measured using a BCA protein assay kit (Thermo Scientific, Rockford, IL, USA). For evaluation of lipid peroxidation in skin homogenates, the malondialdehyde (MDA) content was measured using a thiobarbituric acid reactive substances (TBARS) assay kit (Cayman Chemical, Ann Arbor, MI, USA). The activities of catalase (Cayman Chemical) and glutathione peroxidase (GPx, Cayman Chemical, Ann Arbor, MI, USA) in skin homogenates were measured using assay kits, according to the manufacturer’s instructions.

### 2.11. Quantitative Real-Time Reverse Transcription Polymerase Chain Reaction (RT-PCR)

Total RNA from the skin tissue samples was extracted and real-time RT-PCR was conducted using a Rotor-Gene^TM^ SYBR Green kit (Qiagen, Valencia, CA, USA) and Rotor-Gene 3000 PCR (Corbett Research, Mortlake, Australia), as described previously [28]. The primer sequences used in this study are listed in Table 1. The results were analyzed using the Rotor-Gene 6000 series System Soft program version 6 (Corbett Research). The relative expression of target mRNAs was normalized against the expression of glyceraldehyde 3-phosphate dehydrogenase (Gapdh).

### 2.12. Western Blot Analysis

Dorsal skin tissue samples were lysed as described previously [28]. The protein contents of the skin lysates were measured using a BCA protein assay kit (Thermo Scientific). Western blot analyses were performed as described previously [28]. The antibodies against p-ERK, ERK, p-p38, p38, p-JNK, JNK, c-fos, and β-actin were obtained from Cell Signaling Technology (Beverly, MA, USA). Protein bands were detected using an enhanced chemiluminescence method using Luminata^TM^ Forte Western HRP Substrate (Millipore, Billerica, MA, USA). The relative expression of target protein was estimated using an ImageQuant^TM^ LAS 500 imaging system (GE Healthcare Bio-Science AB, Uppsala, Sweden) and normalized against the expression of β-actin.

### 2.13. Statistical Analysis

All data are expressed as the mean ± SEM. Student’s *t*-test and one-way analysis of variation, followed by Duncan’s multiple comparisons test were carried out using the Statistical Analysis System for Windows version 9.4 (SAS Institute, Cary, NC, USA). Statistical significance was set at *p* < 0.05.

## 3. Results

### 3.1. Phytochemical Content of AB

**Figure 1:**
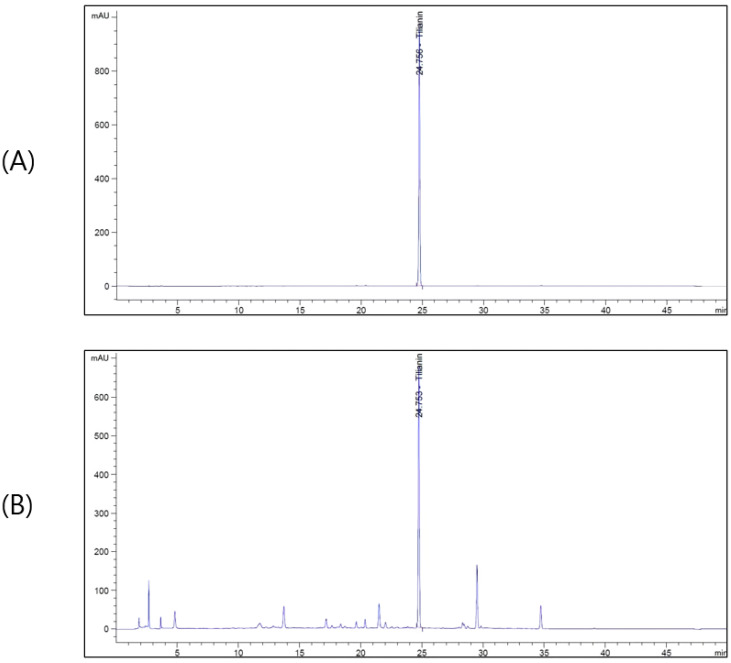
HPLC chromatogram of standard solution (**A**) and AB (**B**).

HPLC analysis was performed to identify Tilianin, a bioactive compound of AB. The developed HPLC method was applied to analyze and identify the bioactive components of AB. The chromatograms are shown in Figure 1. When the content of AB was analyzed, peaks were observed at the retention time of 24.7 min for tilianin at 330 nm.

### 3.2. AB Is More Potent in Increasing the Content of Collagen and Hyaluronic Acid and Decreasing That of MMP-1 in UVB-Irradiated Cells

**Figure 2:**
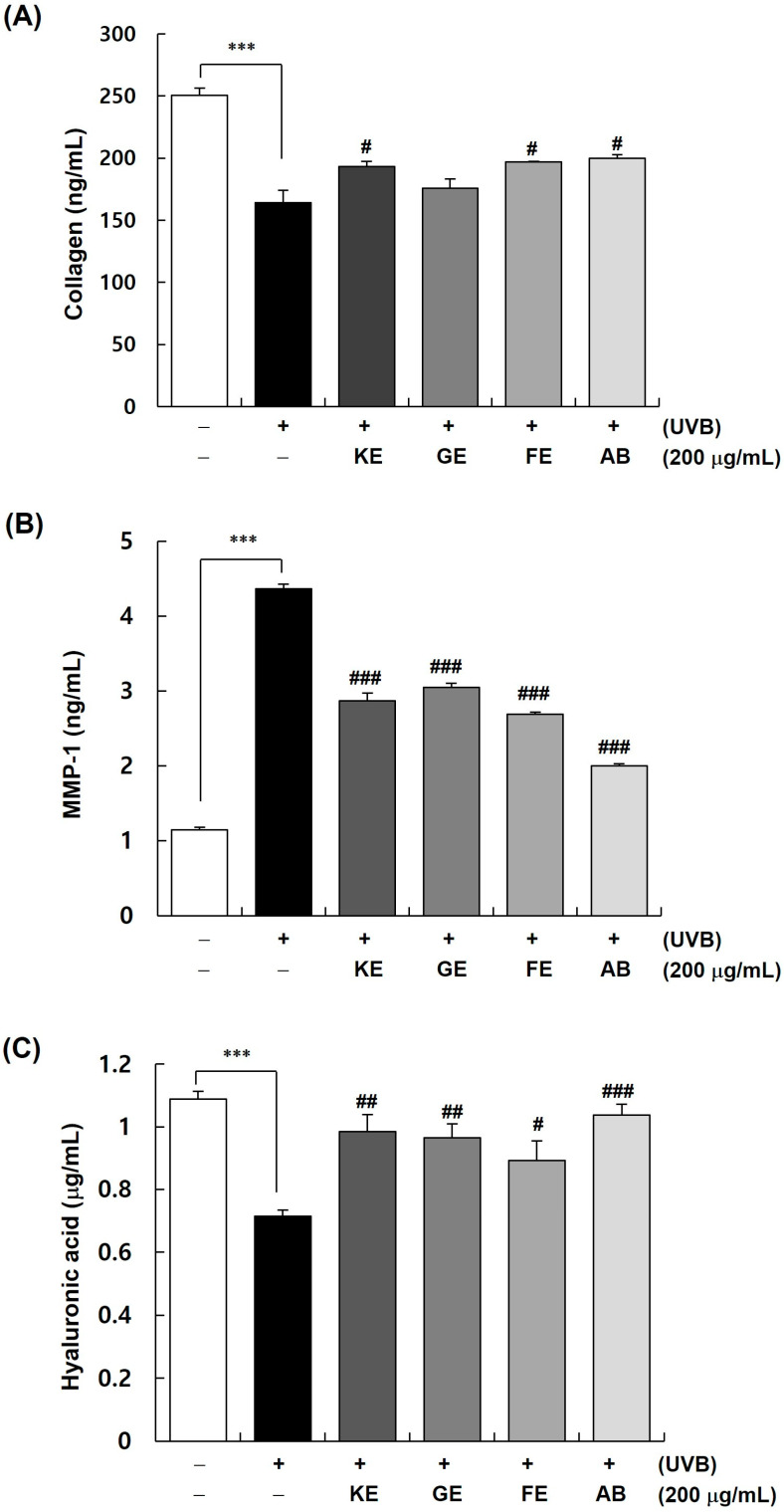
Effect of KE, FE, GE, and AB on the production of collagen, MMP-1, and hyaluronic acid in UVB-irradiated cells. (**A**,**B**) Hs68 cells were plated at 1 × 10^5^ cells/well in 24-well plates and incubated for 24 h. Thereafter, the cells were exposed to UVB irradiation (30 mJ/cm^2^) and incubated in the presence or absence of 200 μg/mL KE, FE, GE, or AB for 24 h. The media conditioned for 24 h were collected. The levels of collagen and MMP-1 in the 24 h-conditioned media were measured. (**C**) HaCaT cells were plated at 1 × 10^5^ cells/well in 24-well plates and incubated for 24 h. Thereafter, the cells were exposed to UVB irradiation (5 mJ/cm^2^) and incubated in the presence or absence of 200 μg/mL KE, FE, GE, or AB for 24 h. The media conditioned for 24 h were collected. The levels of hyaluronic acid in the 24 h conditioned media were measured. Each bar represents the mean ± SEM (*n* = 6). *** *p* < 0.001 indicate significantly different values from that in the [UVB (-)/(-)] group. ^#^ *p* < 0.01, ^##^ *p* < 0.05, ^###^ *p* < 0.001 indicate significantly different values from that in the [UVB (-)/(-)] group.

The collagen content in Hs68 cells irradiated with 30 mJ/cm^2^ UVB in the presence or absence of 200 μg/mL KE, GE, FE, and AB was determined. The collagen content was considerably reduced upon UVB irradiation, but it was significantly increased in cells that were treated with KE and FE (Figure 2A). The content was the highest in the group treated with AB (*p* < 0.05). The effect of each extract on the UVB-stimulated increase in MMP content was determined. UVB irradiation increased the secretion of MMP-1 in the culture supernatant of Hs68 cells by ~3.83-fold, whereas this increase was significantly reduced by the KE, GE, and FE extracts. The inhibitory effect of AB on MMP production was synergistic vis-à-vis the effects of its constituents (Figure 2B). Thus, AB increased the collagen content considerably and effectively decreased the secretion of MMP-1.

We also measured the hyaluronic acid content of HaCaT cells irradiated with 5 mJ/cm^2^ UVB and treated with 200 μg/mL KE, GE, FE, or AB. UVB irradiation reduced the hyaluronic acid content in HaCaT cells, but the content was significantly increased when these cells were treated with KE, GE, or FE extract (Figure 2C); the effect of the AB complex was synergistic with regard to that of its constituents, with the hyaluronic acid content increasing by ~1.46 fold (*p* < 0.05).

### 3.3. AB Alleviates Photoaging on the Dorsal Skin of UVB-Irradiated Hairless Mice

**Figure 3:**
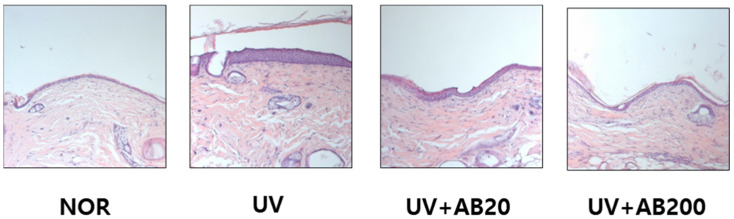
Effect of AB administration on histomorphological changes in the dorsal skin in UVB-irradiated Skh:HR-1 hairless mice. The mice were administered AB through oral gavage for 12 weeks. All mice, except those in the NOR group, were exposed to UVB radiation three times per week for 12 weeks. The dorsal skin tissue was excised, fixed in 4% paraformaldehyde, embedded in paraffin, and sectioned at 5 µm. The tissue sections were stained with H&E. Representative H&E- stained images of dorsal skin tissues (n = 5), 200× magnification.

Dorsal skin tissue was observed under a microscope after H&E staining to investigate histological change caused by UVB irradiation and AB treatment. The skin tissue of the NOR group has a thin epidermal layer with a regular arrangement. Meanwhile, the epidermal layer was remarkably thickened in the UV group compared to the NOR group. The decrease in epidermal layer thickness was observed in the UV+AB20 and UV+UB200 group compared to the UV group (Figure 3).

**Figure 4:**
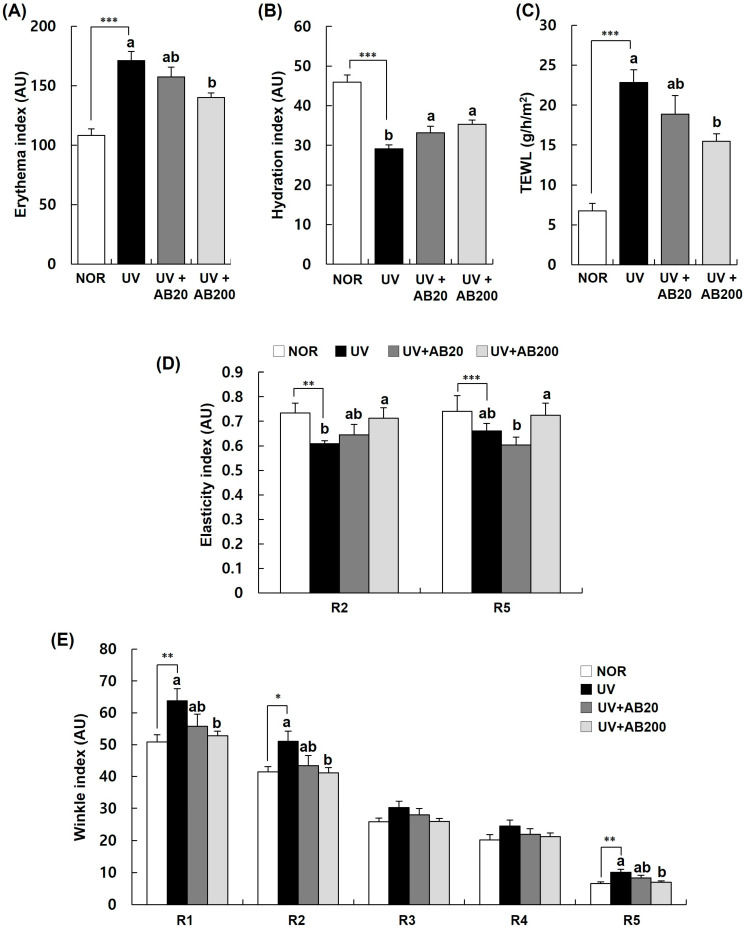
Effect of AB administration on skin indices in UVB-irradiated Skh:HR-1 hairless mice. The mice were administered AB through oral gavage for 12 weeks. All mice, except those in the NOR group, were exposed to UVB radiation three times per week for 12 weeks. Various skin indices were estimated using an individual skin analyzer. (**A**) Erythema index, (**B**) hydration index, (**C**) TEWL, (**D**) elasticity index (R2, gross elasticity; R5, net elasticity), and (**E**) wrinkle index (R1, total roughness; R2, 280 average roughness; R3, maximum roughness; R4, smoothness depth; R5, 281 arithmetic average roughness). Each bar represents the mean ± SEM (*n* = 10). * *p* < 0.01, ** *p* < 0.05, *** *p* < 0.001 indicate significantly different values from that in the NOR group. Different letters indicate significant differences among the UV, UV+AB20, and UV+AB200 groups (*p* < 0.05).

To evaluate whether AB alleviates photoaging induced by UVB irradiation, we measured the changes in five skin-barrier parameters (erythema, moisture content, TEWL, elasticity, and wrinkle index) in irradiated mice and administered vehicle or AB for 12 weeks (Figure 4). The skin erythema index showed a significant improvement in the UVB irradiation group (171.2 ± 7.6 AU) compared with that in the control group (108.4 ± 5.5 AU). Conversely, the AB20 group treated with 20 μg/mL of AB (157.4 ± 8.3 AU) and the AB200 group (140.2 ± 3.7 AU) treated with 200 μg/mL of AB showed a significantly lower erythema index (Figure 4A). The skin moisture index was significantly decreased in the UVB-irradiated group (29.1 ± 1.0 AU) compared with that in the control group (45.9 ± 1.8 AU). However, the skin moisture content in the AB20 (33.2 ± 1.6 AU) and AB200 (35.3 ± 1.1 AU) groups showed a substantial increase (Figure 4B). TEWL, used to evaluate the skin-barrier function, was significantly higher in the UV group (22.8 ± 1.6 g/h/m^2^) than in the NOR group (6.7 ± 1.0 g/h/m^2^). However, TEWL decreased in the AB treatment group, with the decrease being more prominent in the AB200 group (15.5 ± 0.9 g/h/m^2^) compared with that in the AB20 group (18.9 ± 2.3 g/h/m^2^) (Figure 4C).

The skin elasticity index (R2 (total elasticity) and R5 (net elasticity)) in the UV group was lower than that in the NOR group. However, changes in R2 and R5 were significantly improved in the AB200 group compared with that in the UV group. The difference in the AB20 group and UV group was not statistically significant (Figure 4D).

Next, we measured the effect of AB on UVB-induced wrinkle formation on hairless mice (Figure 4E). The value indicating skin roughness (R1) in the UV group was significantly increased to 63.76 ± 3.78 AU. The R1 values in the AB20 and AB200 groups were significantly reduced to 55.71 ± 3.81 and 52.74 ± 1.46 AU, respectively. The average roughness (R2) was 41.4 ± 1.7 and 51.0 ± 3.2 AU in the NOR and UV groups, respectively. In the AB200 group, R2 was significantly reduced (41.1 ± 1.6 AU). There was no statistically significant difference in the maximum roughness (R3) and smoothness depth (R4) between the NOR and UV groups. Moreover, R3 and R4 were not affected by AB treatment. The arithmetic mean roughness (R5) in the UV group (14.1 ± 0.5 AU) was higher than that in the NOR group (9.8 ± 0.5 AU). However, R5 was significantly decreased in the AB200 group compared with that in the UV group (Figure 4E).

### 3.4. AB Increases Hyaluronic Acid and Collagen Expression on the Dorsal Skin of UVB-Irradiated Hairless Mice

**Figure 5:**
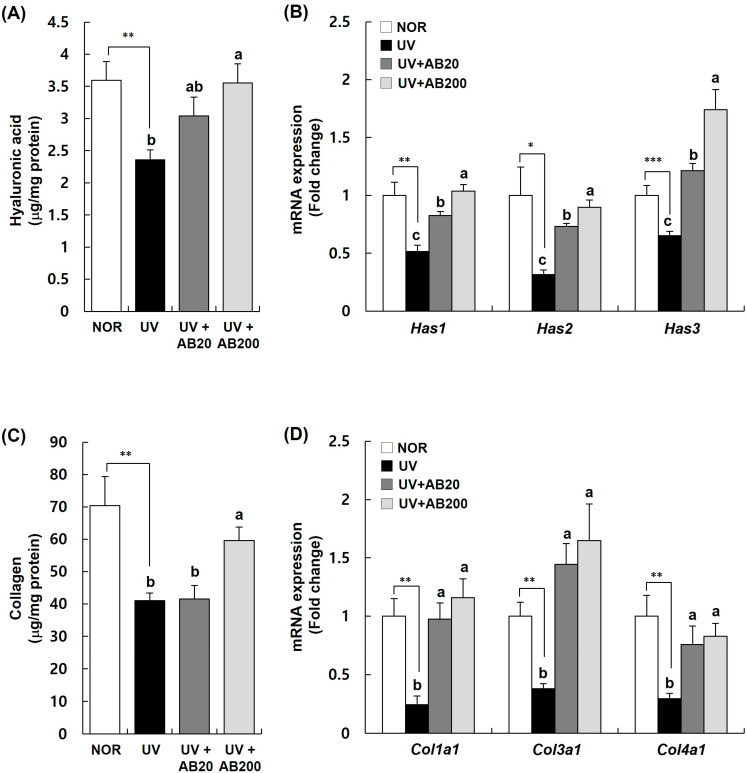
Effect of AB administration on hyaluronic acid and collagen expression in the dorsal skin of UVB-irradiated Skh:HR-1 hairless mice. The mice were administered AB through oral gavage for 12 weeks. All mice, except those in the NOR group, were exposed to UVB radiation three times per week for 12 weeks. (**A**,**C**) The dorsal skin tissue was excised and homogenized. The content of hyaluronic acid and collagen in the skin homogenate was measured using a relevant ELISA kit. (**B**,**D**) Total RNA in the dorsal skin tissue was extracted and reverse-transcribed, and used for real-time PCR. The expression of each mRNA was normalized to that of *Gapdh* and is represented relative to that in the NOR group. Each bar represents the mean ± SEM (*n* = 10). * *p* < 0.01, ** *p* < 0.05, *** *p* < 0.001 indicate significantly different values from that in the NOR group. Different letters indicate significant differences among the UV, UV+AB20, and UV+AB200 groups (*p* < 0.05).

We determined the hyaluronic acid and collagen content of skin homogenates after AB administration in UVB-irradiated mice to evaluate the effect of AB in restoring the skin moisturizing ability and elasticity. The hyaluronic acid content in the UV group was significantly lower than that in the NOR group. On the contrary, this UVB-induced decrease was mitigated by AB. In particular, the hyaluronic acid levels in the AB200 group were significantly increased to 3.55 ± 0.30 μg/mg protein compared with those in the UV group (2.36 ± 0.16 μg/mg protein) (Figure 5A).

Real-time PCR was performed to assess the mRNA expression of hyaluronic acid synthesis genes (*Has1*, *Has2*, *Has3*), which are well-known moisturizing factors. The levels of these mRNAs, which were decreased in the UV group, were significantly increased in a dose-dependent manner by AB. In particular, a remarkable increase in the expression of Has3 mRNA was observed in the AB200 group (Figure 5B).

The effect of AB on collagen content in UVB-irradiated mice is shown in Figure 3C. In the UV group, collagen production in the skin was significantly reduced compared with that in the NOR group. However, the decreased levels in the UV group (41.0 ± 2.3 μg/mg protein) were significantly restored by AB treatment, especially in the AB200 group (59.6 ± 4.2 μg/mg protein) (Figure 5C). Additionally, we assessed the effect of AB on the expression of *Col1a1*, *Col3a1*, and *Col4a1* mRNAs related to collagen synthesis. As shown in Figure 3D, the expression of these genes was significantly increased by AB treatment compared with that in the UV group. In particular, the expression of *Col3a1* mRNA was drastically increased (Figure 5D).

### 3.5. AB Decreases the Expression of MMPs in Dorsal Skin Samples from UVB-Irradiated Hairless Mice

**Figure 6:**
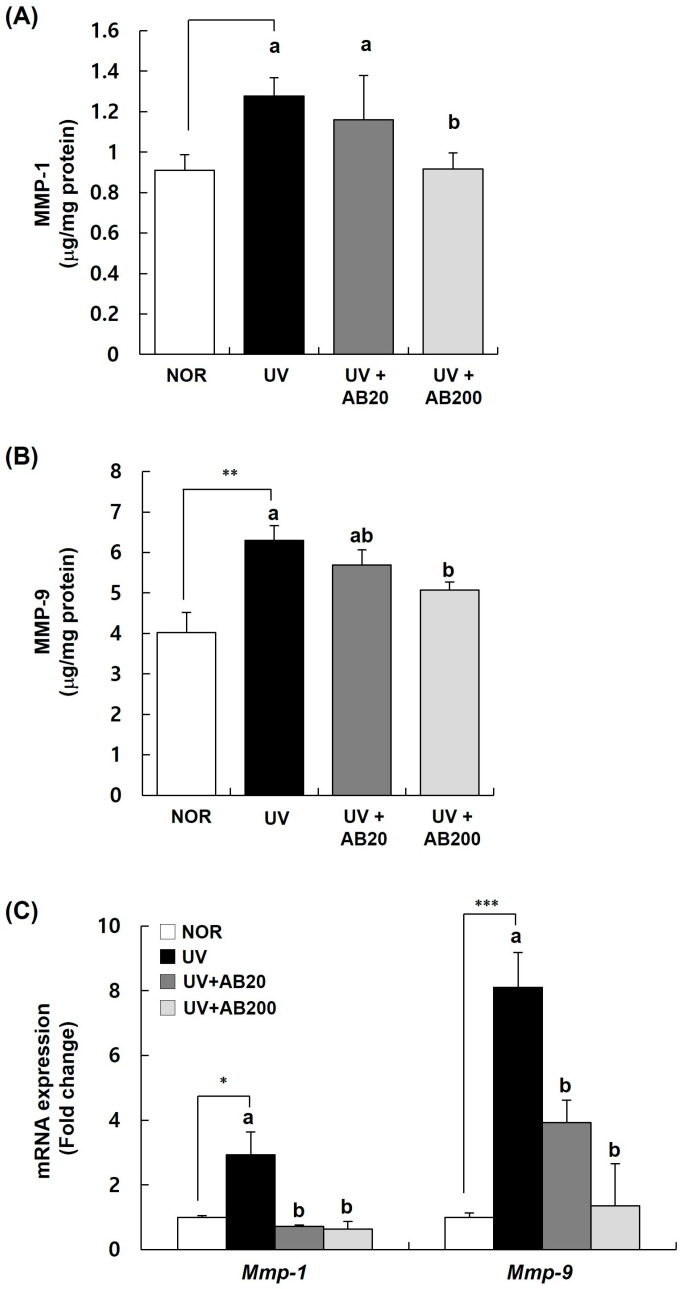
Effect of AB administration on MMP-1 and MMP-9 expression in the dorsal skin of UVB-irradiated Skh:HR-1 hairless mice. The mice were administered AB through oral gavage for 12 weeks. All mice, except those in the NOR group, were exposed to UVB radiation three times per week for 12 weeks. (**A**,**B**) The dorsal skin tissue was excised and homogenized. The content of MMP-1 and MMP-9 in the skin homogenate was measured using a relevant ELISA kit. (**C**) Total RNA in the dorsal skin tissue was extracted and reverse-transcribed and used for real-time PCR. The expression of each mRNA was normalized to that of *Gapdh* and is represented relative to that in the NOR group. Each bar represents the mean ± SEM (*n* = 10). * *p* < 0.01, ** *p* < 0.05, *** *p* < 0.001 indicate significantly different values from that in the NOR group. Different letters indicate significant differences among the UV, UV+AB20, and UV+AB200 groups (*p* < 0.05).

We also analyzed the protein and mRNA levels of MMP-1 and MMP-9 in AB-treated mice to check whether AB has an inhibitory effect on the expression of MMPs induced by UVB irradiation (Figure 6). In the skin tissue, the expression of MMP-1 and MMP-9 was upregulated by UVB irradiation, but was downregulated by AB treatment (Figure 6A,B). In particular, the expression of both MMP-1 and MMP-9 was effectively reduced in the AB200 group. On the contrary, UVB irradiation upregulated the expression of *Mmp-1* and *Mmp-9* mRNA in the skin by approximately 2.93 and 8.10 fold, respectively. These effects were reversed by AB treatment, with both AB20 and AB200 significantly reducing the *Mmp-1* and *Mmp-9* mRNA levels (Figure 6C).

### 3.6. AB Suppresses UVB-Induced MAPK and AP-1 Activation in Dorsal Skin Samples from UVB-Irradiated Hairless Mice

**Figure 7:**
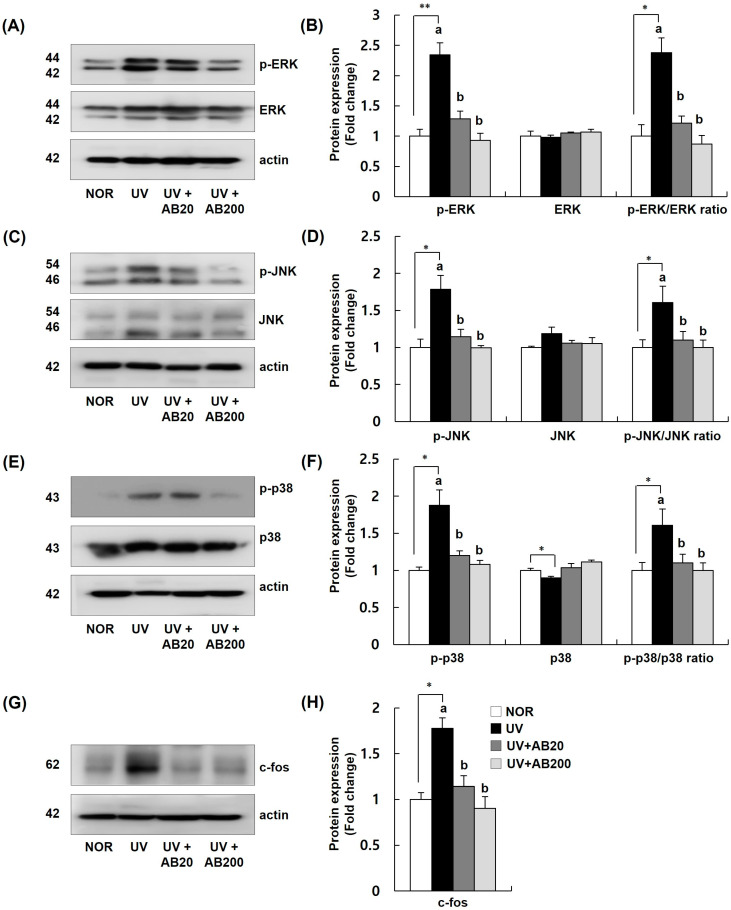
Effect of AB administration on mitogen-activated protein kinase (MAPK) and AP-1 signaling pathway in the dorsal skin of UVB-irradiated Skh:HR-1 hairless mice. The mice were administered AB through oral gavage for 12 weeks. All mice, except those in the NOR group, were exposed to UVB radiation three times per week for 12 weeks. (**A**,**C**,**E**,**G**) Total lysates of the dorsal skin tissue were prepared and analyzed using Western blotting with the indicated antibodies. Images of Western blots representative of three independent experiments are shown. (**B**,**D**,**F**,**H**) Quantitative analysis of Western blot results. The protein expression was normalized to that of β-actin and is represented relative to that in the NOR group. Each bar represents the mean ± SEM (*n* = 10). * *p* < 0.01, ** *p* < 0.05, indicate significantly different values from that in the NOR group. Different letters indicate significant differences among the UV, UV+AB20, and UV+AB200 groups (*p* < 0.05).

The effect of AB on the activation of the MAPK/AP-1 pathway, a key regulator of MMP expression, in UVB-irradiated mice is shown in Figure 7. UVB stimulated the phosphorylation of ERK, JNK, and p38, but this activation was significantly inhibited by AB to a level similar to that in the NOR group. The expression of c-Fos in the skin was significantly increased by UVB irradiation and this increase was mitigated by AB treatment; in the AB200 group, the c-Fos levels were restored to those in the NOR group (Figure 7).

### 3.7. AB Stimulates Antioxidative Enzymes Activities and Reduces Lipid Peroxidation in Dorsal Skin Samples from UVB-Irradiated Hairless Mice

**Figure 8:**
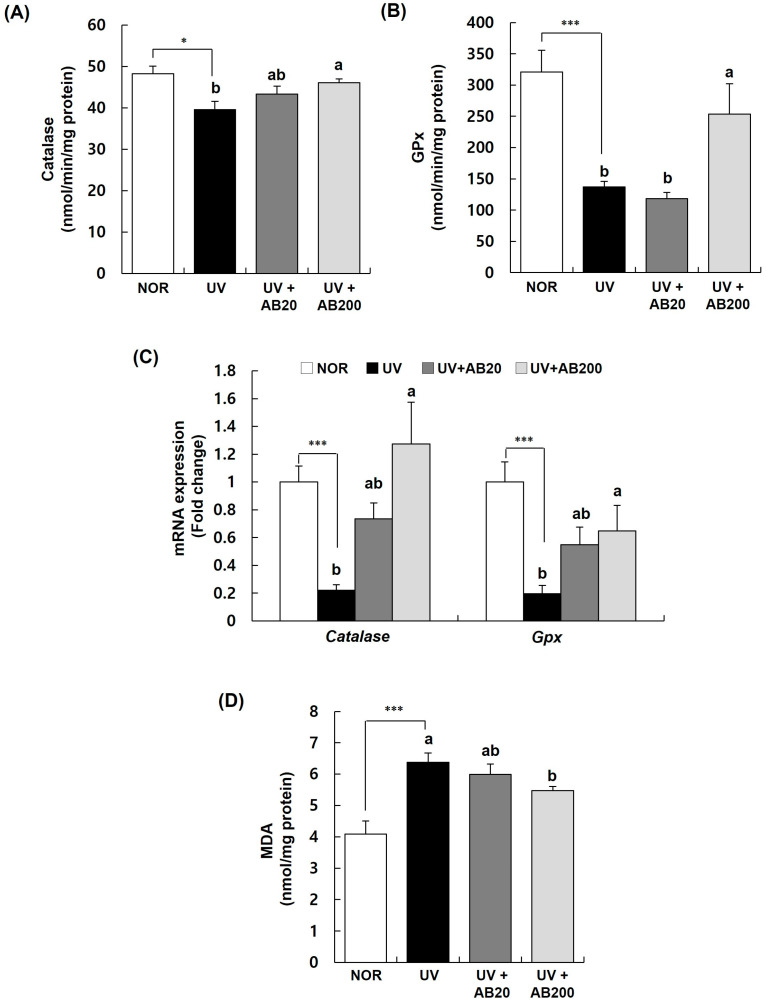
Effect of AB administration on lipid peroxidation and the activities of catalase and glutathione peroxidase (GPx) in the dorsal skin of UVB-irradiated Skh:HR-1 hairless mice. The mice were administered AB through oral gavage for 12 weeks. All mice, except those in the NOR group, were exposed to UVB radiation three times per week for 12 weeks. (**A**,**B**) The dorsal skin tissue was excised and homogenized. The activities of catalase and glutathione peroxidase (GPx) in the skin homogenate were measured using relevant assay kits. (**C**) Total RNA in dorsal skin tissue was extracted and reverse-transcribed and used for real-time PCR. The expression of each mRNA was normalized to that of *Gapdh* and is represented relative to that in the NOR group. (**D**) The dorsal skin tissue was excised and homogenized. The MDA content in the skin homogenate was measured using a TBARS assay kit. Each bar represents the mean ± SEM (*n* = 10). * *p* < 0.01, *** *p* < 0.001 indicate significantly different values from that in the NOR group. Different letters indicate significant differences among the UV, UV+AB20, and UV+AB200 groups (*p* < 0.05).

The expression of antioxidative enzymes in the skin tissue was determined to further investigate whether the protective effect of AB in UVB-irradiated mice was related to the improvement of the antioxidative defense system (Figure 8). The catalase and GPx activities in the skin tissue were downregulated upon UVB irradiation, whereas these were significantly increased by AB, especially in the AB200 group (Figure 8A,B). Similarly, UVB irradiation reduced the expression of *catalase* and *Gpx* mRNAs in the skin to one-fifth of that in the NOR group, whereas AB significantly increased the levels of these mRNAs in a dose-dependent manner (Figure 8C). The MDA content in the skin tissue was significantly increased by UVB irradiation (6.38 ± 0.29 nmol/mg protein) compared with that in the NOR group (4.09 ± 0.42 nmol/mg protein). However, this UVB irradiation-induced increase was significantly suppressed by AB, especially in the AB200 group (5.47 ± 0.14 nmol/mg protein) (Figure 8D).

## 4. Discussion

We made the following six major findings in this study: (1) AB significantly increased the content of collagen and hyaluronic acid and effectively reduced MMP-1 secretion in UVB-irradiated cells, indicating that AB alleviated wrinkle formation and skin moisture loss, which are symptoms of skin photoaging. These results also suggest that AB improves the main cause of wrinkle formation in photoaging by inhibiting the activity of MMPs that destroy collagen; (2) AB significantly improved the skin erythema index and significantly decreased the skin moisture content in UVB-irradiated skin tissues. In addition, the skin wrinkle evaluation factors, R2 (total elasticity) for the skin elasticity index and R1 (skin roughness), R2 (average roughness), and R5 (arithmetic mean roughness) for the skin wrinkle index, were greatly reduced to a level similar to that in the NOR group; (3) AB restored the content of hyaluronic acid and collagen in UVB-irradiated skin tissues, demonstrating enhanced hyaluronic acid structure; (4) AB downregulated the expression of MMP-1 and MMP-9 at both protein and mRNA levels in UVB-irradiated skin tissues; (5) AB inactivated the MAPK and AP-1 pathways in UVB-irradiated skin tissues; (6) AB inhibited the production of MDA, an indicator of oxidative stress, by significantly restoring the antioxidative activity and the expression of antioxidative genes, including catalase and GPx, in UVB-irradiated skin tissue. Therefore, AB can be effective in reversing UVB-induced photoaging by attenuating physical parameters related to photoaging and by regulating collagen turnover and antioxidative-related pathways.

Continuous UVB exposure induces abnormal histological and physiological changes that cause adverse biological effects on the skin, resulting in photoaging symptoms, including wrinkle formation, skin dehydration, skin thickening, and significant loss of elasticity [29,30]. UVB irradiation has been reported to increase epidermal thickness, erythema, and wrinkle parameters in hairless mice [29,31,32]. These results are consistent with the findings of the present study on abnormal physiological changes in UVB-irradiated cells and skin tissues. However, treatment or oral administration of AB alleviated these symptoms induced by UVB irradiation and restored the normal physiological state of the damaged skin, indicating a preventive effect of AB on skin photoaging.

Skin dehydration is another key feature of skin photoaging. UVB irradiation reduced skin hydration and increased TEWL. UVB causes the skin to lose its ability to retain natural moisturizing factors [33]. TEWL is an indicator of the skin-barrier function because epidermis acts as a skin barrier to maintain adequate moisture levels [34]. Because a decrease in water content and an increase in TEWL are accompanied by a decrease in the skin-barrier function, the results of this study suggest that the effect of AB on the recovery of skin moisture is due to its ability to mitigate the UVB-induced damage to the skin-barrier function. Hyaluronic acid, a natural moisturizing factor found abundantly in the dermis, plays an important role in maintaining the skin moisture [35]. Previous studies suggested that repetitive UVB irradiation induces a loss in the hyaluronic acid content due to the reduction in *Has* mRNA expression [17]. Consistent results were found in this study, wherein chronic UVB exposure not only reduced hyaluronic acid levels but also decreased the expression of *Has1*, *Has2*, and *Has3* mRNAs, which was increased by AB treatment. These results indicate that AB has skin moisturizing activity as evidenced by improved TEWL, skin moisture, and hyaluronic acid levels through the upregulation of *Has*.

Collagen is the most abundant protein in the ECM, and the ECM function is highly dependent on the role and structure of collagen [36]. In this study, oral administration of AB significantly increased the collagen content and promoted the expression of *Col1a1*, *Col3a1*, and *Col4a1* mRNAs in the UVB-irradiated skin tissue. These results suggest that AB can effectively prevent the degradation and fragmentation of collagen, and disorganization of collagen fragments, which are the main causes of wrinkle formation in UVB irradiation-induced photoaging. AB also increased the mRNA expression of genes related to skin collagen content and collagen synthesis in the in vivo model of UVB-induced photoaging. We also investigated the molecular mechanisms by which UVB and AB affected the collagen content in the ECM.

Excessive UV irradiation generates ROS, which is a key cellular factor in physiological photoaging [37]. Excessive oxidative stress generated by UVB irradiation induces the activation of the MAPK signaling pathway, followed by that of AP-1 and NF-kappa B. Both these transcription factors independently or cooperatively regulate the expression of target-specific genes involved in a variety of physiological and pathological processes. As oxidative events can modulate AP-1 and NF-kB transcriptional activation, the ability to block UVB-induced AP-1 and NF-κB activation may be due to their antioxidative properties [38]. Moreover, these transcription factors regulate the transcription of genes responsible for ECM degradation, such as MMPs. The activation of the MAPK/AP-1 signaling cascade is a key mechanism in the degradation of generated collagen as well as in the modulation of new collagen formation [39]. The UVB-activated MAPK/AP-1 pathway increases both the mRNA expression and activity of MMPs that degrade elastic fibers and destroy collagen [40]. It is also linked to alteration in numerous molecular signaling pathways in the dermis and epidermis, resulting in the disruption of the antioxidative system, pigmentation, and skin dryness [41]. In this study, AB significantly inhibited the production of MMP-1 in UVB-irradiated fibroblasts, and reduced the expression of MMP-1 and MMP-9 in UVB-irradiated skin tissue at mRNA and protein levels. Compared with the UVB irradiation group, AB also downregulated the protein levels of MAPKs, including p-ERK, p-JNK, and p-p38, and reduced the levels of AP-1 components, such as c-Fos. These results demonstrate that AB alleviates the mRNA expression and activity of MMPs by inactivating the MAPK/AP-1 pathway. Collectively, AB can increase collagen levels in the ECM by improving the collagen turnover-related cell signal transduction in UVB-induced photoaging. Further studies are needed to elucidate whether AB directly interferes with the interaction of MAPK/AP-1 and MMP expression.

We also investigated the effects of AB on lipid peroxidation and on the activity of catalase and GPx. UVB irradiation induces the development of a wide range of skin diseases, such as inflammation, immunosuppression, and tumor promotion [42]. These effects are driven by multiple molecular mechanisms, including DNA damage, lipid peroxidation, and altered enzymatic activity [43]. Recently, Maqui berry (*Aristotelia chilensis*) was reported to clearly reverse UVB irradiation-induced DNA damage by upregulating endogenous cellular enzymes and nonenzymatic antioxidative systems, including superoxide dismutase, catalase, and glutathione, and reducing the production of nitric oxide [44]. Moreover, AB stimulated the activities of antioxidative enzymes and protected against lipid peroxidation in UVB-irradiated skin tissue. This also suggests the molecular mechanisms involved in the protective effect of AB against UVB-induced skin photoaging. Our results indicate that AB can effectively respond to UVB-induced effects by blocking cellular oxidative stress-related events in the skin tissue. In particular, inflammation-related events, such as those on MAPK and AP-1, were significantly affected by AB treatment. Based on these results, it can be hypothesized that AB exerts its effects through an antioxidative mechanism that directly blocks the effects of UV rays or improves the antioxidative cellular network.

Many studies have shown that naturally occurring plant molecules are effective against UV irradiation-induced damage [45,46]. In particular, polyphenol-rich extracts were reported to prevent the damage caused by UV exposure. An extract of *Kaempferia parviflora*, also known as black ginger, which contains a large amount of polymethoxyflavone, increased the expression of collagen synthesis genes compared to that in the UVB control group, significantly hindered wrinkle formation and the loss of collagen fibers, and decreased the expression of MMP through the inhibition of c-Jun and c-Fos activity, and induced catalase expression [32]. Natural compounds from *Eisenia bicyclis*, a type of polyphenol-rich marine brown alga, also inhibited UVB-induced collagen degradation and MMP expression by regulating MAPK/AP-1 signaling, and effectively restored hyaluronic acid production and *Has* mRNA expression [47]. An extract of *Eucalyptus globulus*, an evergreen tree rich in phenolic compounds and gallic acid, inhibited both epidermal changes and collagen degradation, increased skin moisture, and favorably altered the expression of MMP-1, elastin, and procollagen type I proteins [48]. Taken together, these natural plant extracts play a role in protecting against UV-induced photoaging and can be used as medicines, functional foods, and cosmetics [49]. Therefore, AB can be used not only as a functional food ingredient, but also as a cosmetic ingredient such as cream and essence. In particular, the photoprotective properties of AB are considered to be due to the entire plant complex rather than the specific components. Recently, the biological properties of plant complexes composed of several different constituents have been proven to be due to the presence of one or more active constituents, rather than pharmacological effects due to the presence of specific molecules. Rather, the properties could be the combined effects of some or all components of the plant complex.

## 5. Conclusions

In this study, we found that AB prevented wrinkle formation in UVB-irradiated hairless mice by downregulating the MAPK/AP-1 complex signaling pathway that regulates the expression of MMPs. AB increased the collagen content in the dermis by increasing the mRNA expression of collagen and by responding to the regulation of the AP-1 complex. AB also moisturized the skin by stimulating hyaluronic acid formation. Therefore, AB can be a potential antiphotoaging and moisturizing ingredient in skin functional foods. Further clinical studies are needed to determine whether AB is clinically effective as a natural antiphotoaging and moisturizing supplement.

## Figures and Tables

**Table 1:** Sequences of primers used in this study.

Target Gene	Forward Primer (5′-3′)	Reverse Primer (5′-3′)
Catalase	GAACGAGGAGGAGAGGAAAC	TGAAATTCTTGACCGCTTTC
*Col1a1*	GCACGAGTCACACCGGAACT	AAGGGAGCCACATCGATGAT
*Col3a1*	CTAAAATTCTGCCACCCCGAA	AGGATCAACCCAGTATTCTCCACTC
*Col4a1*	CTACGTGCAAGGCAATGAACG	GCAGAACAGGAAGGGCATTGT
Glutathione peroxidase	CGGTTTCCCGTGCAATCAGT	CACCGGGGACCAAATGATG
*Has1*	GTGCGAGTGTTGGATGAAGACC	CACATTGAAGGCTACCCAGTATC
*Has2*	GCCATTTTCCGAATCCAAACAGAC	CCTGCCACACTTATTGATGAGAACC
*Has3*	CTTCAGTCCAGAAACCAAAGTAGG	CTCGTTCCTCAAGAGAAACAAGG
*Mmp-1*	TTGCCCAGAGAAAAGCTTCAG	TAGCAGCCCAGAGAAGCAACA
*Mmp-9*	AGTGGGACCATCATAACATCACAT	TCTCGCGGCAAGTCTTCAG
Gapdh	TGGGTGTGAACCATGAGAAG	GCTAAGCAGTTGGTGGTGC

## Data Availability

The dataset generated during the present study is available upon reasonable request to the corresponding author (E.K.).
